# *MYC* Amplification in Angiosarcoma Arising from an Arteriovenous Graft Site

**DOI:** 10.1155/2015/537297

**Published:** 2015-11-24

**Authors:** Kristen M. Paral, Gordana Raca, Thomas Krausz

**Affiliations:** ^1^Department of Medicine, Section of Dermatology, University of Chicago, 5841 S Maryland Avenue, MC 5067, Chicago, IL 60637, USA; ^2^Department of Medicine, Section of Hematology/Oncology, University of Chicago, 5841 S Maryland Avenue, MC 6092, Chicago, IL 60637, USA; ^3^Department of Pathology, University of Chicago, 5841 S Maryland Avenue, MC 6161, Chicago, IL 60637, USA

## Abstract

Angiosarcoma arising in association with an arteriovenous graft (AVG) or fistula is a unique clinicopathologic scenario that appears to be gaining recognition in the literature. Among reported cases, none has described high-level* MYC* gene amplification, a genetic aberration that is increasingly unifying the various clinicopathologic subdivisions of angiosarcoma. We therefore report the* MYC* gene status in a case of angiosarcoma arising at an AVG site.

## 1. Introduction

Certain predisposing conditions have been clearly associated with angiosarcoma, including (i) radiation exposure, such as ultraviolet light in skin cases and therapeutic irradiation in breast cases; (ii) longstanding lymphedema (i.e., Stewart-Treves syndrome); and (iii) exogenous toxins (e.g., vinyl chloride, thorotrast, and arsenic) in liver cases [1]. Angiosarcoma associated with AVG, however, has not yet achieved the same level of recognition as the aforementioned scenarios. Despite a steady trickle of such cases into the literature over the recent decades [2–11], few authoritative sources acknowledge its existence [12].

An AVG or fistula is an artificial connection between an artery and a vein created with synthetic materials such as polytetrafluoroethylene (PTFE) or Dacron [13]. Implanted foreign material has been linked to the development of sarcoma for many decades [14]. Most cases of angiosarcoma associated with an AVG have been linked to immunosuppression, although other etiological factors have been suggested, such as the alteration of blood flow [2, 11]. Minimal attention has been devoted to the role of genetic aberrations in these cases.

Recent findings of frequent high-level* MYC* gene amplification in angiosarcomas suggest a genetic basis for the disease [15]. The* MYC* gene is a potent oncogene that is amplified in various clinicopathologic subdivisions of angiosarcoma, including secondary (postirradiation and lymphedema-associated) and a subset of primary angiosarcomas (breast, skin, and bone) [15–19].* MYC* amplification status has not yet been described in the unusual setting of AVG-associated angiosarcoma; we therefore report* MYC* amplification status in one such case.

## 2. Case Report

A 63-year-old Hispanic man presented in 2013 for evaluation of left arm pain in an area where an AVG had been placed 15 years previously (a brachiocephalic “loop graft” with PTFE). The AVG had not been in use for eight years owing to a kidney transplant in 2005. Prior to the transplant, the patient required regular hemodialysis due to end-stage renal disease from chronic hypertension and diabetes.

Physical examination revealed swelling and tenderness of the AVG site, which was attributed to probable thrombosis. This was pursued by angiogram, which was interpreted as a thrombosed pseudoaneurysm at the site. The angiogram also disclosed an unexpected finding: a destructive mass involving the elbow. Further imaging with MRI studies revealed a 20 cm mass encircling the elbow and tracking distally into the forearm. The mass was biopsied to show angiosarcoma, resulting in transhumeral amputation. Six months after the amputation, the patient is alive without evidence of sarcoma.

### 2.1. Pathology

**Figure 1:**
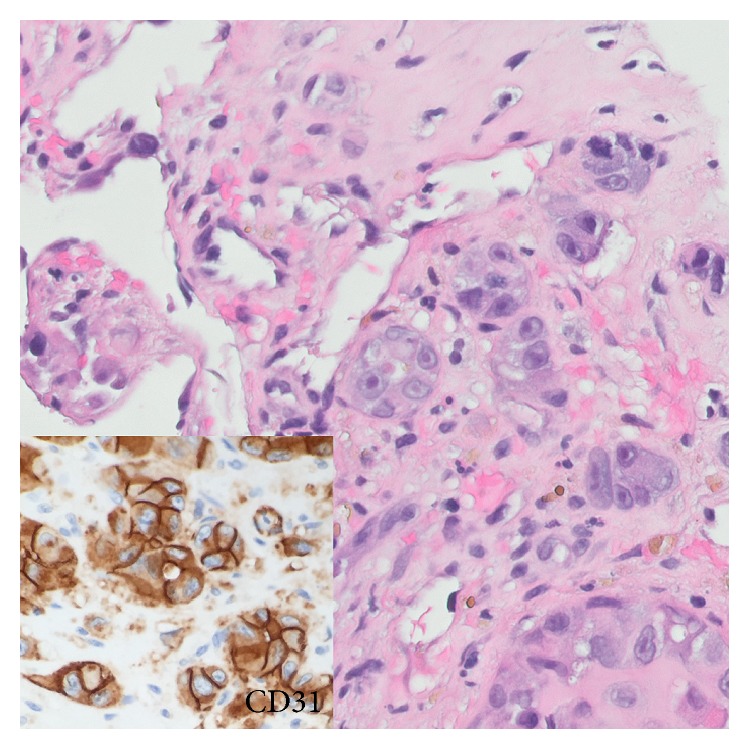
Microscopic examination, biopsy material. The conspicuous clusters of malignant epithelioid cells dominate the picture and could give rise to a broad differential diagnosis that includes carcinoma and melanoma. The poorly formed vascular channels in the background could easily be overlooked. Immunohistochemistry was key (inset).

**Figure 2:**
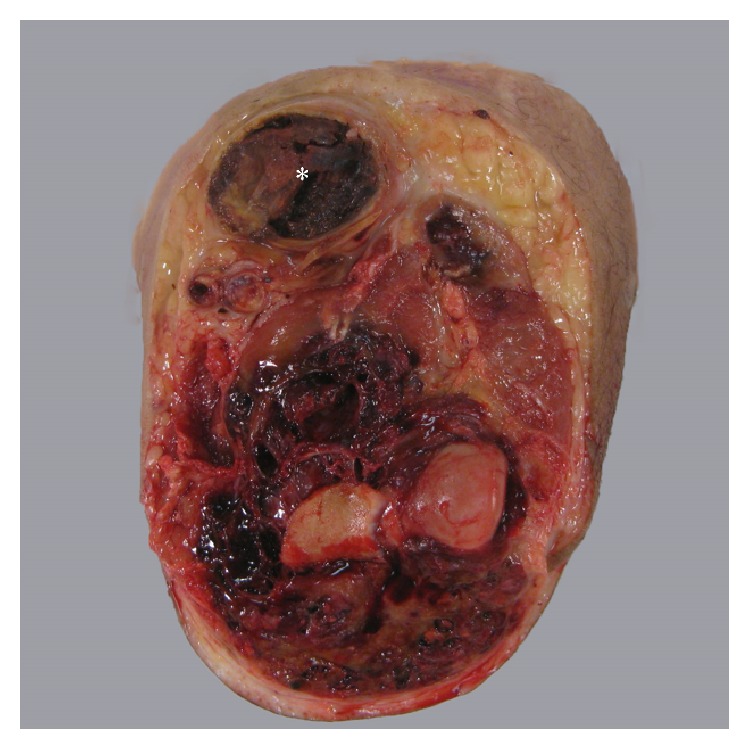
Gross examination, amputation specimen. The AVG site (asterisk) in the antecubital region exhibits a dilated space distended with tumor, which radiologically was thought to be thrombus within a pseudoaneurysm. Tumor envelopes the elbow and tracks distally into the forearm.

**Figure 3:**
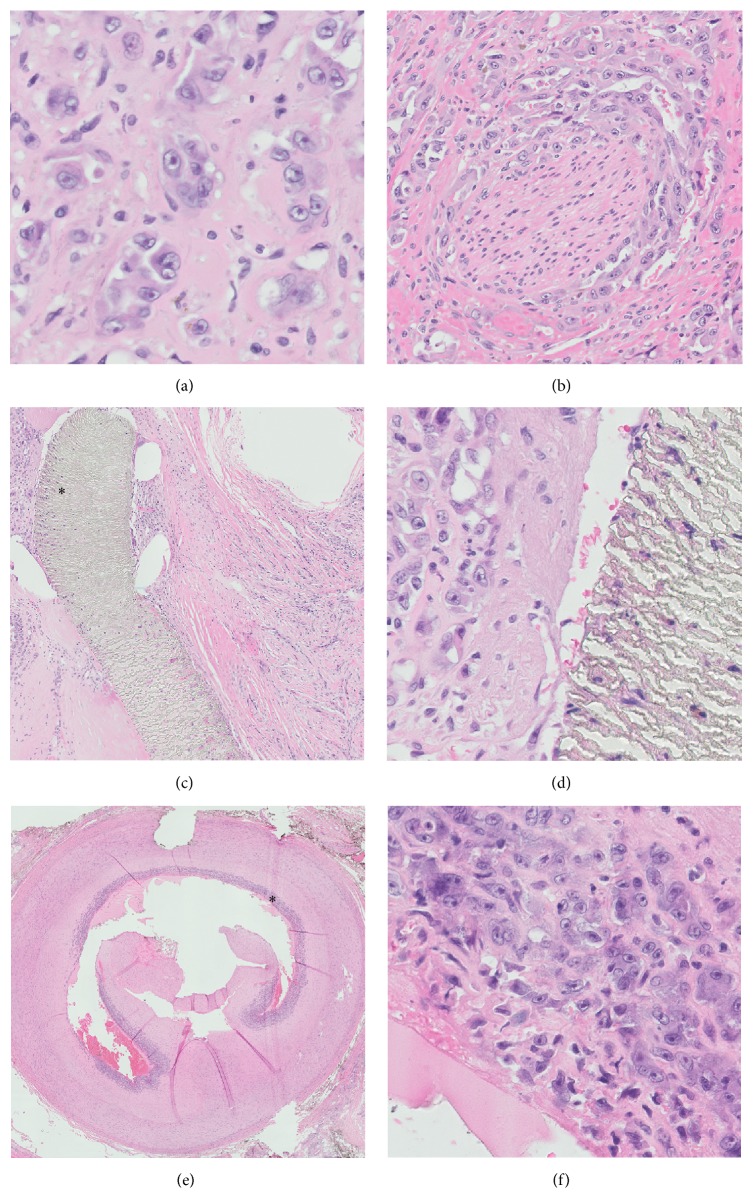
Microscopic examination, amputation specimen. (a) Clusters of malignant epithelioid cells, similar to those seen in the biopsy specimen, are noted (original magnification ×400). (b) Perineural invasion can explain the patient's pain symptoms (original magnification ×200). (c) Tumor clusters are embedded within the dense fibrous sheath around the graft material; the region marked by the asterisk is shown at higher power in (d) (original magnification ×50, ×400). (e) Tumor cells are seen tracking along the intimal layer of a large artery; the region marked by the asterisk is shown at higher power in (f) (original magnification ×10, ×400).

Initial biopsy of the mass disclosed clusters of malignant epithelioid cells adjacent to poorly formed vascular spaces that showed strong, diffuse expression of vascular markers by immunohistochemistry (Figure 1). Patchy cytokeratin expression paralleled the epithelioid morphology (CK AE1/AE3, CK7, and CK8/18). The subsequent amputation specimen showed tumor at the AVG site (Figure 2). Microscopic examination showed predominantly epithelioid cells with minimal spindle cell morphology and near-absent vasoformation; therefore, a final diagnosis of epithelioid angiosarcoma was rendered. Among the most interesting findings were (1) the presence of tumor juxtaposed to foreign graft material and (2) the finding of tumor tracking along a vessel wall. Two lymph nodes recovered near the resection margin showed metastatic angiosarcoma. The salient histopathologic findings are shown in Figure 3.

### 2.2. Molecular Findings

**Figure 4:**
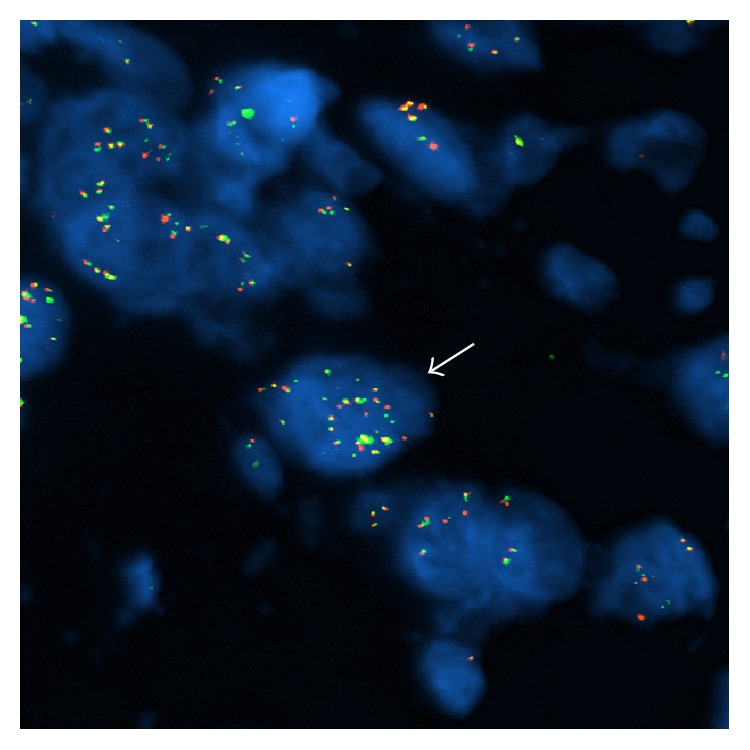
FISH results using a* MYC* break-apart probe. Representative tumor cell (indicated by arrow) is showing an increased number of fusion signals (more than 20), indicative of high-level amplification of the* MYC* locus.

FISH analysis for the quantitation of* MYC* was performed on formalin-fixed, paraffin-embedded tissue sections cut at 5 mm using commercially available break-apart FISH probe-set for the* MYC* locus (8q24), with the 5′ probe labeled with Spectrum Red and the 3′ probe labeled with Spectrum Green (Abbott Molecular, Des Plaines, IL, USA). With this probe-set a yellow fusion signal is produced by the juxtaposition of the 5′ and the 3′ probe when the* MYC* locus has a normal configuration. Standard laboratory protocols and quality control measures were followed for this study. In each case, 100 interphase nuclei were analyzed in a blinded manner by two technicians (200 total nuclei). Amplification of the* MYC* locus was defined as the presence of increased number of* MYC* signals (more than 6) in tumor cells. Clear high-level* MYC* amplification was seen in large areas, with an average of more than 20 signals per cell (Figure 4).

## 3. Discussion

The finding of* MYC* amplification in this case of AVG-associated angiosarcoma expands the spectrum of recognized clinicopathologic subtypes of angiosarcoma bearing this aberration and suggests they share similar pathogenesis. The present case exhibited large regions of clear signal amplification by FISH, consistent with the most commonly observed pattern described in the literature for angiosarcoma [15]. One of the many effects of* MYC* gene amplification is augmentation of a cell's preexisting gene expression program [20] and promotion of angiogenesis [15]. The presence of* MYC* amplification in this case does not necessarily exclude a pathogenic role for the synthetic graft material. There is evidence to suggest that the fibrous capsule that develops around foreign material may stimulate carcinogenesis by providing a home-base for macrophages, which secrete cytokines, growth factors, and free radicals that can induce genetic damage, even after fibrosis is complete [14]. Perhaps this mechanism can account for* MYC* gene aberration in foreign body-associated angiosarcoma. Although the fibrous capsule in this case was fully appreciated, documentation of fibrous encapsulation tends to be inconsistently reported [21].

Alternatively, the graft material may itself directly provoke angiogenesis and subsequent tumorigenesis. Indeed, newer-generation “biopolymers” seek to incorporate bioactive molecules that directly stimulate vascular regeneration [13]. Further studies will be necessary to investigate this and other possible contributing factors, such as immunosuppression. A link to immunosuppression in this case could not be established because the patient was partially managed by an external institution. In any event, the true underlying mechanism for tumorigenesis in this case and in many cases of angiosarcoma likely involves a tangled web of interactions between genetic changes and microenvironment alterations.
